# DKK3 expression is associated with immunosuppression and poor prognosis in glioblastoma, in contrast to lower-grade gliomas

**DOI:** 10.1186/s12883-023-03236-0

**Published:** 2023-05-06

**Authors:** Myung-Hoon Han, Jeong Min Baek, Kyueng-Whan Min, Jin Hwan Cheong, Je Il Ryu, Yu Deok Won, Mi Jung Kwon, Seong-Ho Koh

**Affiliations:** 1 Department of Neurosurgery, Hanyang University Guri Hospital, Hanyang University College of Medicine, Guri, Gyeonggi-do Republic of Korea; 2 Department of Translational Medicine, Graduate School of Biomedical Science & Engineering, Hanyang University, Hanyang University, Seoul, South Korea; 3 Department of Pathology Uijeongbu Eulji Medical Center, Eulji University School of Medicine, Uijeongbu, Gyeonggi-do Republic of Korea; 4 Department of Pathology, Hallym University Sacred Heart Hospital, Hallym University College of Medicine, Anyang, Gyeonggi-do Republic of Korea; 5 Department of Neurology, Hanyang University Guri Hospital, 11923 Gyeongchun-ro, Guri-Si, Gyeonggi-do Republic of Korea

**Keywords:** Glioblastoma multiforme, Lower grade glioma, Wnt/β-catenin signaling, Dickkopf-3, Survival, Disease progression

## Abstract

**Purpose:**

We previously reported that expression of dickkopf-3 (DKK3), which is involved in the Wnt/β-catenin pathway, is significantly associated with prognosis in patients with glioblastoma multiforme (GBM). The aim of this study was to compare the association of DKK3 with other Wnt/β-catenin pathway-related genes and immune responses between lower grade glioma (LGG) and GBM.

**Methods:**

We obtained the clinicopathological data of 515 patients with LGG (World Health Organization [WHO] grade II and III glioma) and 525 patients with GBM from the Cancer Genome Atlas (TCGA) database. We performed Pearson’s correlation analysis to investigate the relationships between Wnt/β-catenin-related gene expression in LGG and GBM. Linear regression analysis was performed to identify the association between DKK3 expression and immune cell fractions in all grade II to IV gliomas.

**Results:**

A total of 1,040 patients with WHO grade II to IV gliomas were included in the study. As the grade of glioma increased, DKK3 showed a tendency to be more strongly positively correlated with the expression of other Wnt/β-catenin pathway-related genes. DKK3 was not associated with immunosuppression in LGG but was associated with downregulation of immune responses in GBM. We hypothesized that the role of DKK3 in the Wnt/β-catenin pathway might be different between LGG and GBM.

**Conclusion:**

According to our findings, DKK3 expression had a weak effect on LGG but a significant effect on immunosuppression and poor prognosis in GBM. Therefore, DKK3 expression seems to play different roles, through the Wnt/β-catenin pathway, between LGG and GBM.

**Supplementary Information:**

The online version contains supplementary material available at 10.1186/s12883-023-03236-0.

## Introduction

Gliomas are the most common malignant primary brain tumors in adults [1]. They are diffuse infiltrative tumors that cause significant morbidity and mortality. Gliomas are classified as grade I, II, III, and IV according to their histological characteristics such as the mitotic index, necrosis, microvascular proliferation, and endothelial proliferation [2].

It is well-documented that tumor cells can exhibit stem cell-like properties, and cancer stemness is known as the most fundamentally important characteristic of malignancy [3]. A large amount evidence suggests that suppression of immune responses from the tumor microenvironment is related to a poor prognosis in the majority of cancers [4]. Cancer stemness is not only a fundamental process of tumor progression but also closely related to antigenicity, intratumoral heterogeneity, and immune suppression in cancer [3]. It is well-established that glioblastoma multiforme (GBM) cancer stem cells induce treatment resistance and an immunosuppressive GBM microenvironment [5–7]. The Wnt/β-catenin signaling pathway is known to play a crucial role in the progression of gliomas and maintenance of glioma stem cells by inhibiting tumor cell differentiation [8–10]. Therefore, we previously identified a significant gene, *dickkopf-3 (DKK3)*, belonging to the Wnt/β-catenin pathway that is associated with immunosuppression in GBM [11]. DKK3 expression was significantly associated with mortality and disease progression in patients with GBM.

As a common malignant brain tumor, LGG progresses to GBM within 5–10 years. During this process, the expression of many genes and molecular pathways changes [12]. Methylation of the O-6-methylguanine-DNA methyltransferase (MGMT) promoter, the promoter for TERT (which encodes telomerase), isocitrate dehydrogenase 1 (IDH1), and codeletion of chromosome arms 1p and 19q (1p/19q codeletion) are important markers for the molecular classification of gliomas [13, 14]. Therefore, it is important to identify novel genetic and molecular markers for the diagnosis and treatment of gliomas of different grades [15]. Through a comparative analysis of differentially expressed genes between different grades of gliomas, we may enhance our knowledge of the occurrence, development, and transformation mechanisms behind different malignant brain tumors.

Therefore, we subsequently wanted to investigate whether DKK3 expression is also associated with immunosuppression and prognosis in lower grade glioma (LGG) using the Cancer Genome Atlas (TCGA) database in this study. Another goal of this study was to compare the association of DKK3 with other significant genes related to Wnt/β-catenin signaling and immune responses between LGG and GBM. Therefore, based on the results of this study, we wanted to determine how DKK3 could act on the Wnt/β-catenin signaling pathway in LGG and GBM to significantly affect mortality and disease progression in patients with GBM.

## Materials and methods

### Study patients

We recently performed studies using 525 cases of GBM with available virtual histopathological slides, clinical information, and mRNA expression information obtained from the TCGA database (https://gdc.cancer.gov/about-data/publications/pancanatlas and https://www.cbioportal.org/) [11, 16]. To determine whether there is a difference in the role of the *DKK3* gene between LGG and GBM, 515 cases of LGG (World Health Organization [WHO] grade II and III glioma) were additionally obtained from the TCGA database. We were able to obtain information on the mRNA expression of over 20,000 genes in LGG and clinical information such as the period to disease progression and death, WHO glioma grade (grade II or III), histological type (astrocytoma type, oligodendroglioma type, or oligoastrocytoma type), presence of an IDH1 mutation, Karnofsky Performance Scale Index, and radiation treatment from the TCGA database. Therefore, a total of 1,040 WHO grade II to IV gliomas were finally included in the study. Although grade III gliomas are high-grade gliomas, according to the National Institutes of Health official website as well as several previous studies using TCGA data, grades II and III gliomas have been defined as LGGs (https://www.cancer.gov/ccg/research/genome-sequencing/tcga/studied-cancers/lower-grade-glioma-study) [17, 18]. In addition, as described in the above-mentioned TCGA studies, patients with oligodendrogliomas were included in the LGG group in this study. However, because oligodendrogliomas differ from astrocytomas in terms of cellular structure and histopathology, we performed an additional analysis that excluded patients with oligodendrogliomas from the LGG group. The raw data of our study can be found in Supplementary Data 1.

#### Informed consent

was not required because the data were obtained from the publicly available TCGA database.

### Gene set related to the Wnt/β-catenin pathway

The Molecular Signatures Database (MSigDB version 7.5.1) in the Gene Set Enrichment Analysis (GSEA) (version 4.3.2) (https://www.gsea-msigdb.org/) was used to obtain the gene set associated with the Wnt/β-catenin pathway (standard name, ST_WNT_BETA_CATENIN_PATHWAY; systematic name, M17761) [11, 16]. From the Wnt/β-catenin-related gene set, four genes (*AXIN2, NKD1, NKD2*, and *RPSA*) were not available in the TCGA database of GBM (Supplementary Data 1). Therefore, we included a total of 30 genes related to the Wnt/β-catenin pathway: *AKT1, AKT2, AKT3, ANKRD6, APC, AXIN1, CBY1, CER1, CSNK1A1, CTNNB1, CXXC4, DACT1, DKK1, DKK2, DKK3, DKK4, DVL1, FRAT1, FSTL1, GSK3A, GSK3B, LRP1, MVP, PIN1, PSEN1, PTPRA, SENP2, SFRP1, TSHB*, and *WIF1*. We extracted mRNA expression data for these 30 genes from the TCGA database of 515 and 525 cases of LGG and GBM, respectively (Supplementary Data 1).

### ***In silico*** flow cytometry

Tumor-infiltrating lymphocytes in glioma tissues were analyzed using CIBERSORT (https://cibersort.stanford.edu), which is a versatile computational method for quantifying the immune cell-type fractions based on a validated leukocyte gene signature matrix containing 547 genes and 22 human immune cell subpopulations [19]. The gene expression profiles of glioma tissues were entered into CIBERSORT, and the algorithm was run using the LM22 signature matrix at 100 permutations.

CD8 + T cells are considered major drivers of antitumor immunity [20]. Elevated CD8 + T-cell counts in the tumor microenvironment is positively associated with a good prognosis in cancer [21, 22]. In addition, CD4 + T cells, CD8 + T cells, regulatory T cells (Tregs), B cells, and antigen-presenting cells (APCs) are known to play a crucial role in the GBM immune microenvironment [23, 24]. Therefore, as in our previous study, we included the following eight representative immune cells to investigate the relationship between DKK3 expression and the degree of antitumor immunity in both grade II or III glioma and the GBM microenvironment: CD8 + T cells, regulatory T cells, naive CD4 + T cells, resting and activated memory CD4 + T cells, memory B cells, plasma B cells, and activated dendritic cells [11].

### Preparation of human brain tumor tissue samples

Whole human brain tissue lysates from healthy adults were obtained from Novus Biologicals (Littleton, CO, USA). Glioma tissue samples were obtained from patients who underwent surgical resection at the Department of Neurosurgery of Hanyang University Guri Hospital, Korea, in November 2016 [25]. Freshly resected tumor tissues were collected during surgery and immediately submitted to the laboratory for storage at -80 °C in a nearby facility.

The study protocol was reviewed and approved by the Institutional Review Board of Guri Hospital (IRB No. 2016-10-002) and adhered to the tenets of the Declaration of Helsinki. All patients provided written informed consent prior to participating in the study.

### Western blot analysis

DKK3 expression levels in normal human brains and collected brain tumor tissues were confirmed by western blot analysis. Briefly, glioma tissue samples were washed twice with 4℃ D-PBS, and lysis buffer was added (RIPA II cell lysis buffer 1X with Triton, without ethylene-diamine-tetraacetic acid [EDTA]; 1 mM sodium orthovanadate (Na3VO4); 1 mM phenylmethyl-sulfonyl fluoride [PMSF]; 1 mM sodium fluoride [NaF]; protease inhibitor cocktail 1X; and 1 mM EDTA, pH 8.0). The tissues were then sonicated (Sonoplus; Bandelin Electronics, Berlin, Germany) and incubated in an ice bucket for 30 min. Prior to western blotting, protein assays were performed to confirm the protein concentrations in the tumor and normal human brain lysates (Novus Biologicals, Colorado, USA) using bicinchoninic acid solution (Sigma, Missouri, USA). We used equal concentrations of protein (10 µg); samples were separated using 4 ~ 12% sodium dodecyl sulfate-polyacrylamide gel electrophoresis (SDS-PAGE, Invitrogen, Waltham, USA). Proteins were then transferred onto polyvinylidene fluoride membranes (Millipore, Bedford, MA, USA). The membranes were blocked with 2% skimmed milk for 1 h and incubated with primary antibodies against DKK3 (1:1000, Abcam, UK) and GAPDH (1:4000, Cell Signaling, Minnesota, USA). After washing the membranes with Tris-buffered saline (TBS, 1X) containing 0.1% Tween-20 (TBST), they were incubated with HRP-conjugated anti-rabbit antibodies (1:2000, Jackson ImmunoResearch Laboratories, Pennsylvania, USA). The bands were visualized using the West-Q Chemiluminescent Substrate Kit (GenDEPOT, Texas, USA) and an image analyzer (ImageQuant LAS 4000; GE Healthcare, Little Chalfont, UK).

### Statistical analysis

The chi-square test and Student’s *t*-test were used to assess differences in variables between grade II or III glioma and GBM groups. Heatmap analyses of Wnt/β-catenin-related gene expression and immune cell infiltration between all grade II to IV glioma tissues were conducted using the “pheatmap” package of R software (version 4.1.2). Box plots were used to visualize differences in the expression of the genes and degree of immune cell infiltration between all grade II to IV gliomas.

Pearson’s correlation coefficients and significance levels were estimated to investigate the relationships between Wnt/β-catenin-related gene expression and between DKK3 expression and the degree of immune cell infiltration in both grade II or III glioma and GBM. To visualize the correlations, we used the “corrplot” package of R software with the clustering technique (R code: corrplot, M, order = “hclust”, p.mat = p_mat, sig.level = 0.01, method = “square”).

A scatterplot with a linear regression line was used to visualize the association between Wnt/β-catenin-related significant gene expression and between DKK3 expression and the degree of immune cell infiltration in all grade II to IV gliomas. We additionally used locally weighted scatter plot smoothing (LOWESS) to graphically represent the overall relationship between DKK3 expression and the degree of immune cell infiltration in all grade II–IV glioma tissues.

We calculated the overall survival (OS) and progression-free survival (PFS) rates using Kaplan–Meier analysis based on gene expression tertiles (tertile 1 = low expression; tertile 2 = moderate expression; tertile 3 = high expression).

A p-value < 0.05 was considered statistically significant. All statistical analyses were performed using R software version 4.1.2 and SPSS for Windows version 24.0 (IBM, Chicago, IL).

## Results

### Characteristics of the study patients

A total of 1,040 patients with WHO grade II to IV gliomas from the TCGA database were included in the study (Table 1). There were 515 patients with WHO grade II or III gliomas and 525 patients with WHO grade IV GBM. The mean patient age at diagnosis of glioma was 50.4 years, and 41.8% of patients were female. A total of 731 (70.3%) patients underwent radiation treatment. Further detailed information is shown in Table 1.

**Table 1:** Clinical characteristics of study patients with glioma

Characteristics	Grade II or III glioma	GBM	Total	p
Number, (%)	515 (49.5)	525 (50.5)	1040	
Sex, female, n (%)	230 (44.7)	205 (39.0)	435 (41.8)	0.067
Age at diagnosis of glioma, mean ± SD, y	42.9 ± 13.4	57.7 ± 14.6	50.4 ± 15.8	< 0.001
Time between glioma diagnosis and death (months), mean ± SD	31.7 ± 31.5	17.0 ± 18.0	24.3 ± 26.6	< 0.001
Time between glioma diagnosis and disease progression (months), mean ± SD	25.8 ± 25.7	10.2 ± 13.0	17.9 ± 21.7	< 0.001
WHO-grade glioma, n (%)				N/A
Grade II	250 (48.5)	N/A	250 (24.0)	
Grade III	265 (51.5)		265 (25.5)	
Grade IV	N/A	525 (100)	525 (50.5)	
Glioma histological type, n (%)				N/A
Astrocytoma	194 (37.7)	N/A	194 (18.7)	
Oligodendroglioma	191 (37.1)		191 (18.4)	
Oligoastrocytoma	130 (25.2)		130 (12.5)	
Glioblastoma	N/A	525 (100)	525 (50.5)	
IDH1 mutation, n (%)				< 0.001
Yes	91 (17.7)	14 (2.7)	105 (10.1)	
No	34 (6.6)	230 (43.8)	264 (25.4)	
Missing data	390 (75.7)	281 (53.5)	671 (64.5)	
Karnofsky Performance Scale Index, median (IQR)	80.0 (80.0–90.0)	80.0 (70.0–80.0)	80.0 (80.0–90.0)	< 0.001
Missing data, n (%)	52 (10.1)	133 (25.3)	185 (17.8)	
Radiation treatment, n (%)				< 0.001
Yes	296 (57.5)	435 (82.9)	731 (70.3)	
No	185 (35.9)	70 (13.3)	255 (24.5)	
Missing data	34 (6.6)	20 (3.8)	54 (5.2)	

GBM, glioblastoma multiforme; SD, standard deviation; WHO, World Health Organization; IDH1, isocitrate dehydrogenase; IQR, interquartile range

### Differences in Wnt/β-catenin-related gene expression between grade II or III glioma and GBM

**Fig. 1:**
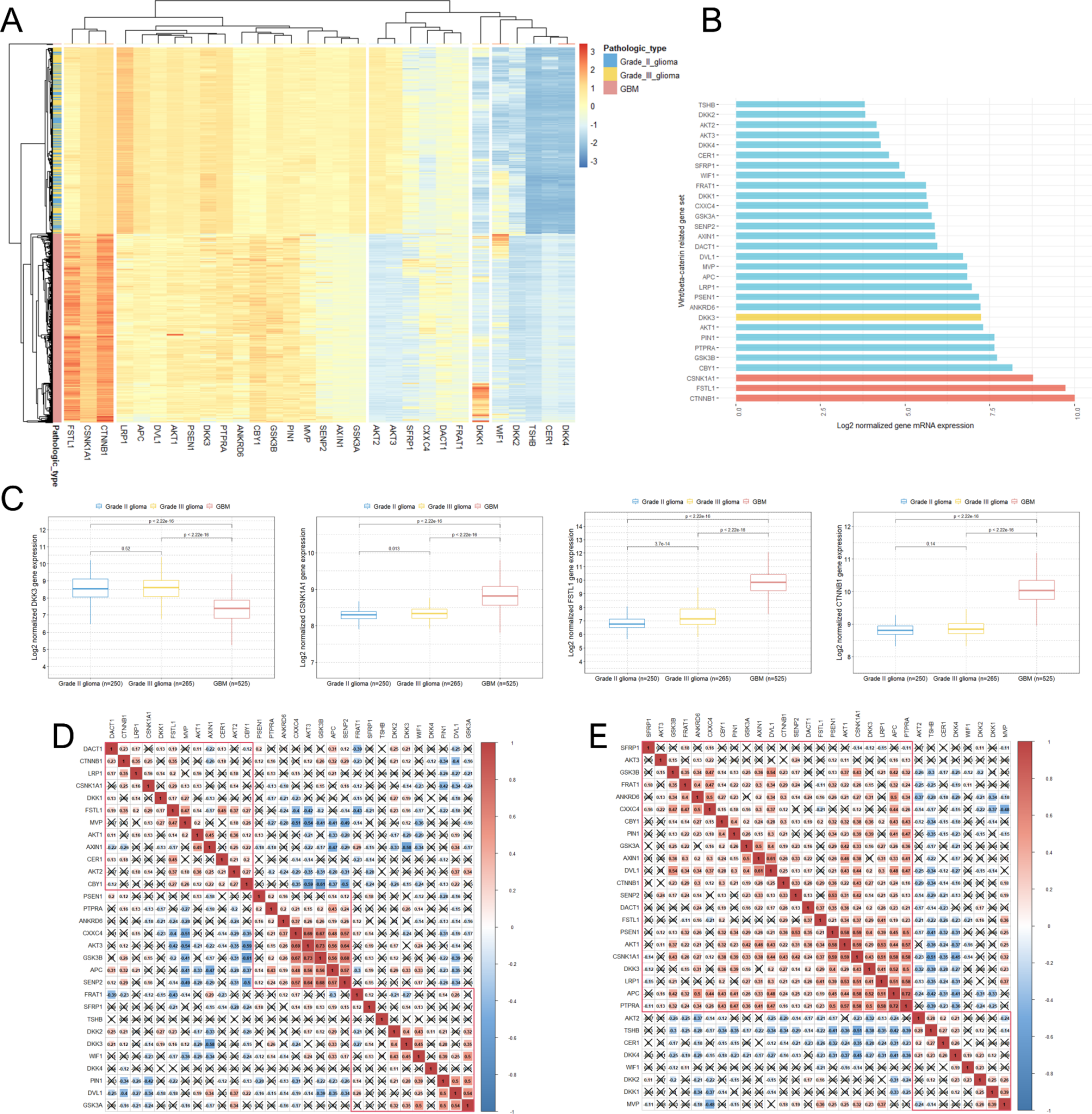
Wnt/β-catenin pathway-related gene expression patterns in patients with grade II to IV glioma. **(A)** A hierarchically clustered heatmap showing the expression patterns of the 30 genes related to the Wnt/β-catenin signaling pathway in patients with grade II to IV glioma. Gene expression levels were log2 transformed, and color density indicating the levels of log2 fold changes is presented. Red and blue represent up- and downregulated expression, respectively, in grade II to IV glioma; **(B)** Bar plots indicating average gene expression levels related to the Wnt/β-catenin pathway in GBM tissue; **(C)** Boxplots showing the differences in DKK3, CSNK1A1, FSTL1, and CTNNB1 expression levels according to the WHO grade of glioma; **(D)** Pearson correlation coefficients and significance levels were calculated between Wnt/β-catenin pathway-related genes in WHO grade II and III glioma; **(E)** Pearson correlation coefficients and significance levels were calculated between the Wnt/β-catenin pathway-related genes in WHO grade IV GBM. The color-coordinated legend indicates the value and sign of Pearson’s correlation coefficient. The number in the box indicates Pearson’s correlation coefficient. The x in the box indicates a p-value ≥ 0.001 *CSNK1A1*, casein kinase 1 alpha 1; *CTNNB1*, catenin beta 1; *DKK3*, dickkopf Wnt signaling pathway inhibitor 3; *FSTL1*, follistatin-like 1; GBM, glioblastoma multiforme

The heatmap showed log2-transformed fold changes (deviation from the median expression) in Wnt/β-catenin pathway-related gene expression across grade II to IV glioma tissues (Fig. 1A). The overall pattern of Wnt/β-catenin pathway-related gene expression was relatively homogeneous between grade II and grade III glioma. However, we observed clear differences in Wnt/β-catenin pathway-related gene expression between grade II or III glioma and GBM. Interestingly, there were Wnt/β-catenin-related genes whose mRNA expression levels were noticeably increased in GBM compared with grade II and III glioma, and those genes were catenin beta 1 (CTNNB1), follistatin-like 1 (FSTL1), and casein kinase 1 alpha 1 (CSNK1A1) (Fig. 1B **and C)**. However, when comparing the DKK3 mRNA expression levels between grade II or III glioma and GBM, we found that DKK3 expression was significantly decreased in GBM compared with grade II or III glioma (Fig. 1C). We further investigated the correlations between the mRNA expression levels of all Wnt/β-catenin-related genes in both grade II or III glioma and GBM (Fig. 1D **and E)**. Overall, significant positive correlations between Wnt/β-catenin-related genes were significantly increased in GBM compared with grade II and III glioma. (Fig. 1D **and E)**.

### Correlation between DKK3 and immune cells in grade II or III glioma and GBM

**Fig. 2:**
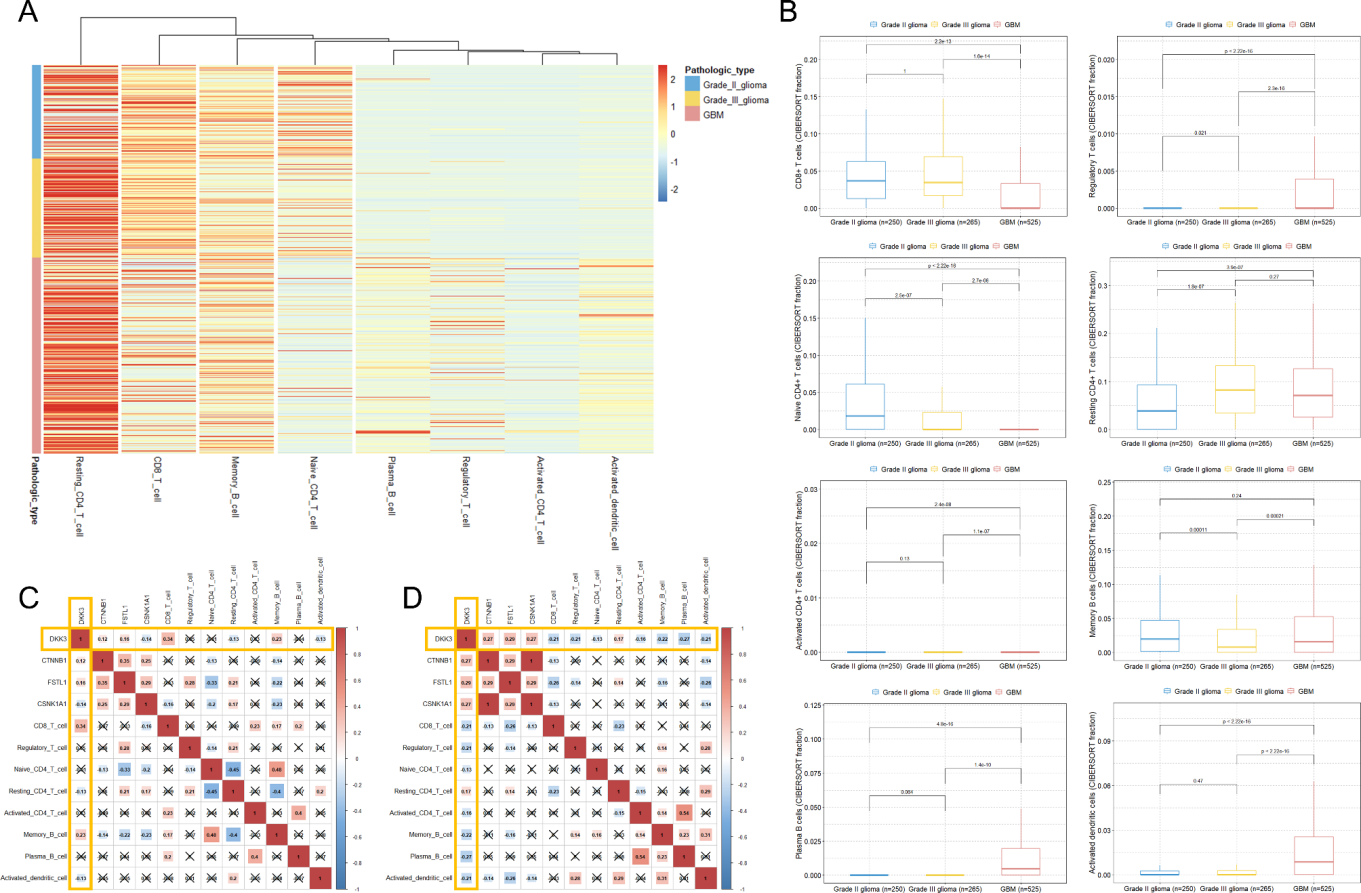
Comparison of immune cell fractions between grade II and IV glioma and the correlations between DKK3, CSNK1A1, FSTL1, and CTNNB1 gene expression and fractions of representative immune cells. **(A)** A hierarchically clustered heatmap showing the expression patterns of eight representative immune cells according to the WHO grade of glioma; **(B)** Boxplots showing the differences in eight representative immune cell fractions according to the WHO grade of glioma; **(C)** Pearson correlation coefficients and significance levels were calculated between DKK3, CSNK1A1, FSTL1, and CTNNB1 gene expression and the cell fractions of eight representative immune cells in WHO grade II and III glioma; **(D)** Pearson correlation coefficients and significance levels were calculated between DKK3, CSNK1A1, FSTL1, and CTNNB1 gene expression and the cell fractions of eight representative immune cells in WHO grade IV GBM. The color-coordinated legend indicates the value and sign of Pearson’s correlation coefficient. The number in the box indicates Pearson’s correlation coefficient. The x in the box indicates a p-value ≥ 0.001 *CSNK1A1*, casein kinase 1 alpha 1; *CTNNB1*, catenin beta 1; *DKK3*, dickkopf Wnt signaling pathway inhibitor 3; *FSTL1*, follistatin-like 1; GBM, glioblastoma multiforme

The heatmap showed differences in immune cell fractions between grade II to IV glioma tissues (Fig. 2A). The CD8 + T-cell and naive CD4 + T-cell fractions were significantly lower in GBM than in grade II and III glioma (Fig. 2B). On the other hand, the fractions of regulatory T cells, activated CD4 + T cells, plasma B cells, and activated dendritic cells were significantly increased in GBM compared with grade II and III glioma. We calculated the correlations between the expressions levels of the DKK3, CTNNB1, FSTL1, and CSNK1A1 genes and eight immune cell fractions in grade II or III glioma and GBM (Fig. 2C **and D)**. In grade II and III glioma, DKK3 showed a significant positive correlation with CD8 + T cells and memory B cells (Fig. 2C). However, in grade IV GBM, DKK3 showed statistically significant correlations (p < 0.001) with all eight immune cell fractions (an x in the box indicates a p-value ≥ 0.001) (Fig. 2D). Interestingly, DKK3 was significantly negatively correlated with all immune cells except for the resting CD4 + memory T-cell fraction in GBM.

### Associations between selected genes involved in the Wnt/β-catenin pathway

**Fig. 3:**
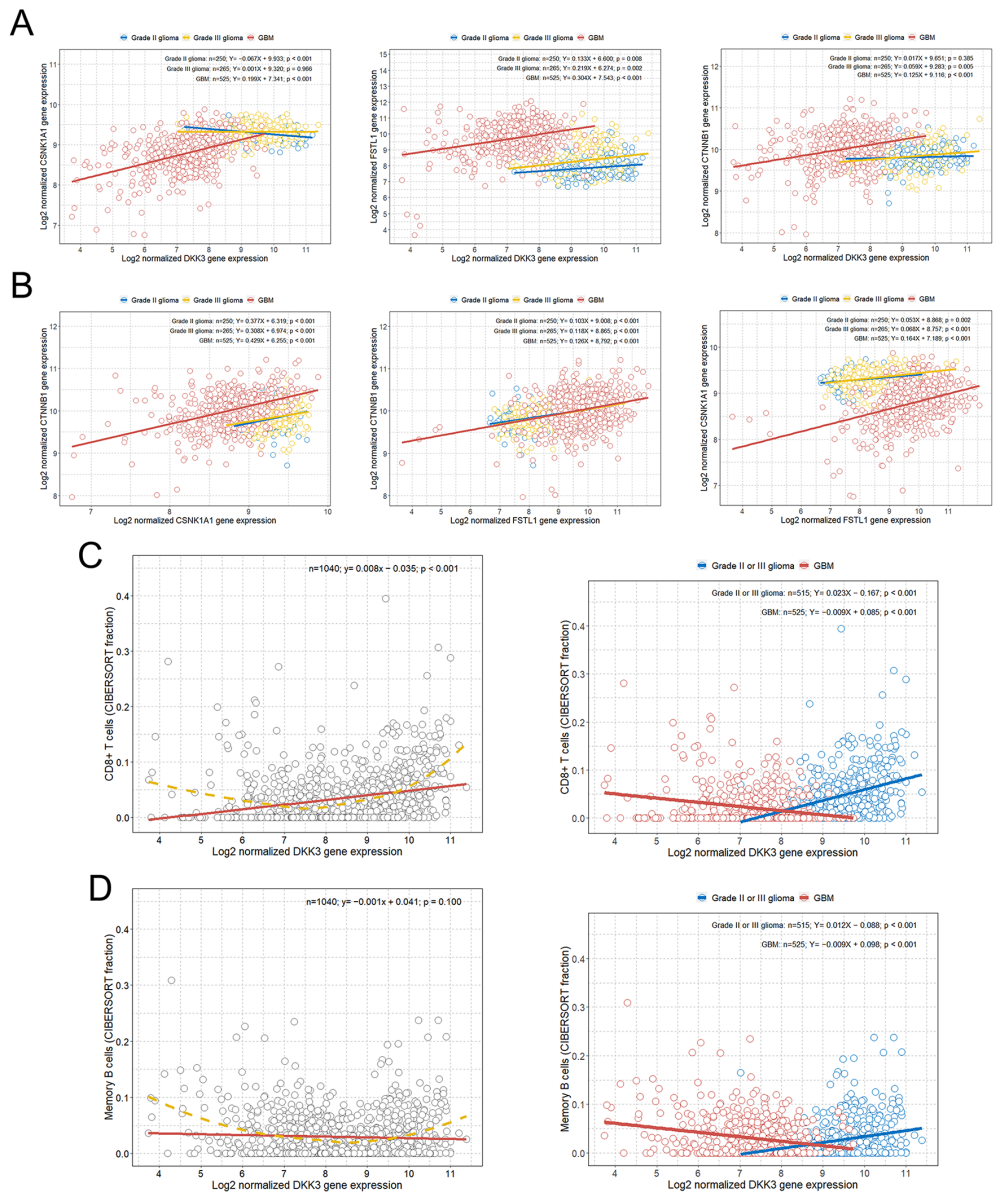
Scatter plot with linear regression line between Wnt/β-catenin-related significantly expressed genes and between DKK3 expression and immune cell fractions in all grade II to IV gliomas. **(A)** Scatter plot with linear regression lines showing the association between DKK3 and CSNK1A1, FSTL1, and CTNNB1 gene expression based on the WHO grade of glioma; **(B)** Scatter plot with linear regression lines showing the association among CSNK1A1, FSTL1, and CTNNB1 gene expression based on the WHO grade of glioma; **(C)** (Left) Scatterplot with a linear regression line and LOWESS curve showing the overall relationship between DKK3 expression and the CD8 + T-cell fractions in all grade II to IV gliomas; (Right) Scatter plot with linear regression lines showing the associations between DKK3 expression and the CD8 + T-cell fractions according to WHO grade II or III glioma and GBM; **(D)** (Left) Scatterplot with a linear regression line and LOWESS curve showing the overall relationship between DKK3 expression and the memory B-cell fractions in all grade II to IV gliomas; (Right) Scatter plot with linear regression lines showing the associations between DKK3 expression and the memory B-cell fractions according to WHO grade II or III glioma and GBM. *CSNK1A1*, casein kinase 1 alpha 1; *CTNNB1*, catenin beta 1; *DKK3*, dickkopf Wnt signaling pathway inhibitor 3; *FSTL1*, follistatin-like 1; GBM, glioblastoma multiforme

We identified linear associations between the expression of the DKK3, CTNNB1, FSTL1, and CSNK1A1 genes classified by the WHO glioma grade. When the relationship between DKK3 and CSNK1A1 was calculated, a significant negative association was shown between DKK3 and CSNK1A1 in grade II glioma (Fig. 3A). However, in grade III glioma, the relationship between DKK3 and CSNK1A1 was not statistically significant, and in grade IV GBM, the association between DKK3 and CSNK1A1 showed a significant positive correlation (Fig. 3A). When determining the relationships among DKK3, FSTL1 and CTNNB1, we observed that the higher the grade of glioma was, the stronger the positive correlations between DKK3 and FSTL1 and between DKK3 and CTNNB1 (Fig. 3A). On the other hand, when the relationships between CSNK1A1 and CTNNB1 and between FSTL1 and CTNNB1 were estimated, positive correlations with similar slopes were shown in both grade II or III glioma and GBM (Fig. 3B). However, when the relationship between FSTL1 and CSNK1A1 was estimated, the positive correlation between FSTL1 and CSNK1A1 was stronger in GBM than in grade II or III glioma (Fig. 3B).

### Differences in the association between DKK3 and immune cells between grade II or III glioma and GBM

We observed an overall significant positive association between DKK3 expression and the CD8 + T-cell fraction with a LOWESS curve showing a curvilinear relationship in all grade II to IV gliomas (Fig. 3C). When the patients were divided into grade II or III glioma and GBM groups, a significant positive correlation was found between DKK3 expression and the CD8 + T-cell fraction in the grade II or III glioma group. On the other hand, in the GBM group, a significant negative correlation between DKK3 expression and the CD8 + T-cell fraction was observed (Fig. 3C). In addition, when evaluating the relationship between DKK3 and memory B cells in all patients with glioma, the LOWESS curve also showed a curvilinear relationship (Fig. 3D). However, when the patients were classified by grade II or III glioma and GBM, the results were similar to the abovementioned relationship between DKK3 and CD8 + T cells. A significant positive correlation was observed between DKK3 expression and the memory B-cell fraction in the grade II or III glioma group, whereas in the GBM group, a significant negative correlation was found between DKK3 expression and the memory B-cell fraction (Fig. 3D).

### Associations between DKK3, CTNNB1, FSTL1, and CSNK1A1 expression and OS and PFS in patients with glioma

**Fig. 4:**
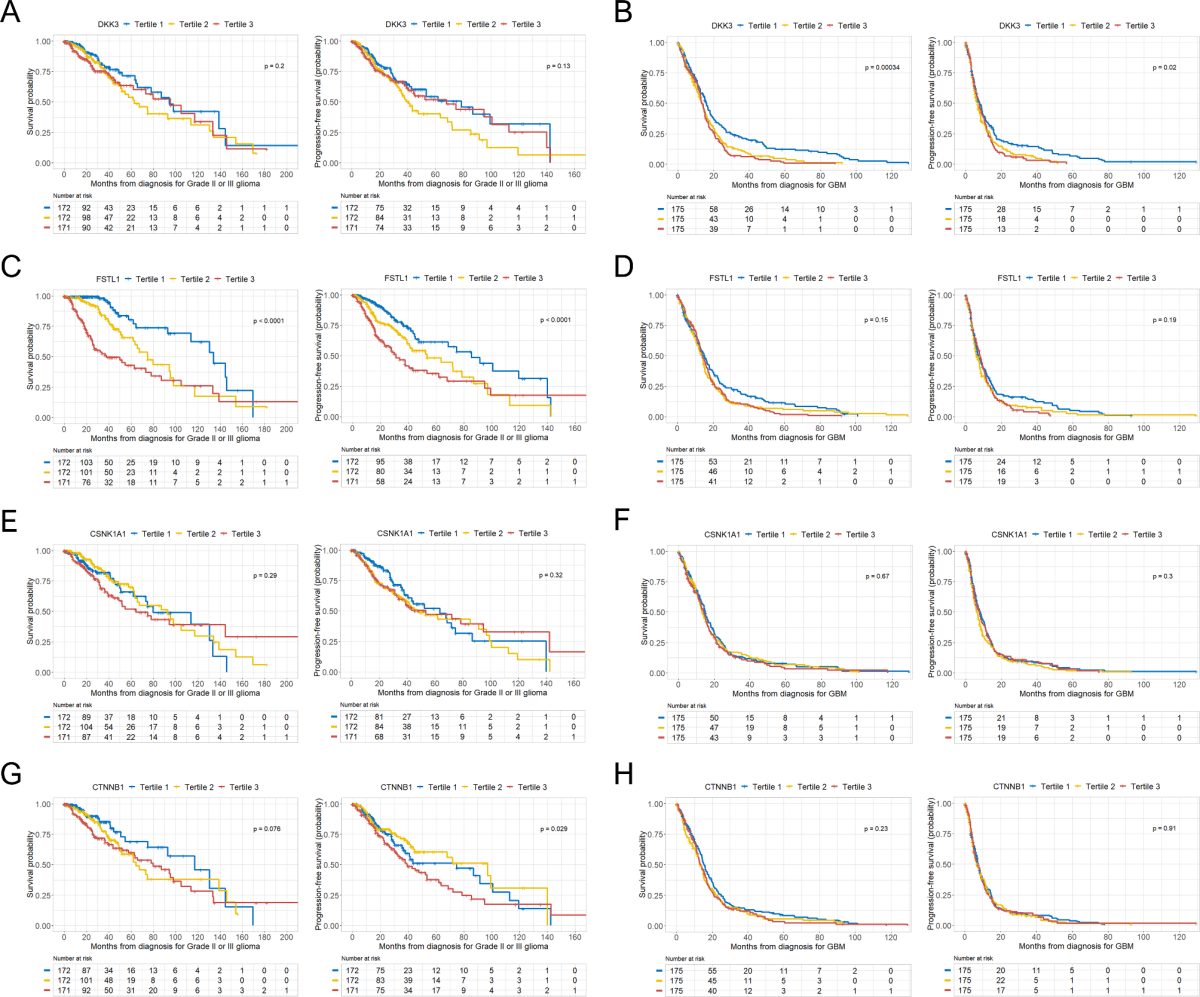
OS and PFS rates according to the expression of DKK3, CSNK1A1, FSTL1, and CTNNB1 in patients with grade II or III glioma and GBM. **(A)** Kaplan–Meier curves showing the OS and PFS rates according to DKK3 tertiles in WHO grade II or III glioma; **(B)** Kaplan–Meier curves showing the OS and PFS rates according to DKK3 tertiles in WHO grade IV GBM; **(C)** Kaplan–Meier curves showing the OS and PFS rates according to FSTL1 tertiles in WHO grade II or III glioma; **(D)** Kaplan–Meier curves showing the OS and PFS rates according to FSTL1 tertiles in WHO grade IV GBM; **(E)** Kaplan–Meier curves showing the OS and PFS rates according to CSNK1A1 tertiles in WHO grade II or III glioma; **(F)** Kaplan–Meier curves showing the OS and PFS rates according to CSNK1A1 tertiles in WHO grade IV GBM; **(G)** Kaplan–Meier curves showing the OS and PFS rates according to CTNNB1 tertiles in WHO grade II or III glioma; **(H)** Kaplan–Meier curves showing the OS and PFS rates according to CTNNB1 tertiles in WHO grade IV GBM. *CSNK1A1*, casein kinase 1 alpha 1; *CTNNB1*, catenin beta 1; *DKK3*, dickkopf WNT signaling pathway inhibitor 3; *FSTL1*, follistatin-like 1; GBM, glioblastoma multiforme; OS, overall survival; PFS, progression-free survival

The Kaplan-Meier survival curves for OS and PFS in patients with grade II or III glioma and GBM are presented according to the tertile groups of gene expression of DKK3, CTNNB1, FSTL1, and CSNK1A1 in Fig. 4. Although not statistically significant in patients with grade II or III glioma, patients with GBM in the first DKK3 tertile showed significantly greater OS and PFS rates than those in the second and third tertiles (Fig. 4A **and B)**. Conversely, the first FSTL1 tertile showed significantly greater OS and PFS rates than the second and third tertiles only among patients with grade II or III glioma (Fig. 4C **and D)**. When OS and PFS were additionally calculated for the CSNK1A1 and CTNNB1 tertile groups in patients with grade II or III glioma and GBM, the first CTNNB1 tertile group showed a significantly higher PFS rate among patients with grade II or III glioma, while the rest were not statistically significantly different (Fig. 4E-H).

We also present the results of the analysis that excluded patients with oligodendrogliomas from the LGG group in Supplementary Fig. 1. We observed that there were statistically significant differences in OS according to the different expression levels of DKK3 and CTNNB1 in the LGG group excluding patients with oligodendrogliomas compared with those in the LGG group including patients with oligodendrogliomas (Supplementary Fig. 1). However, overall, there were no significant differences in the results between the two LGG groups.

### Hypothetical role of DKK3 in glioma progression considering its interaction with CTNNB1, FSTL1, and CSNK1A1

**Fig. 5:**
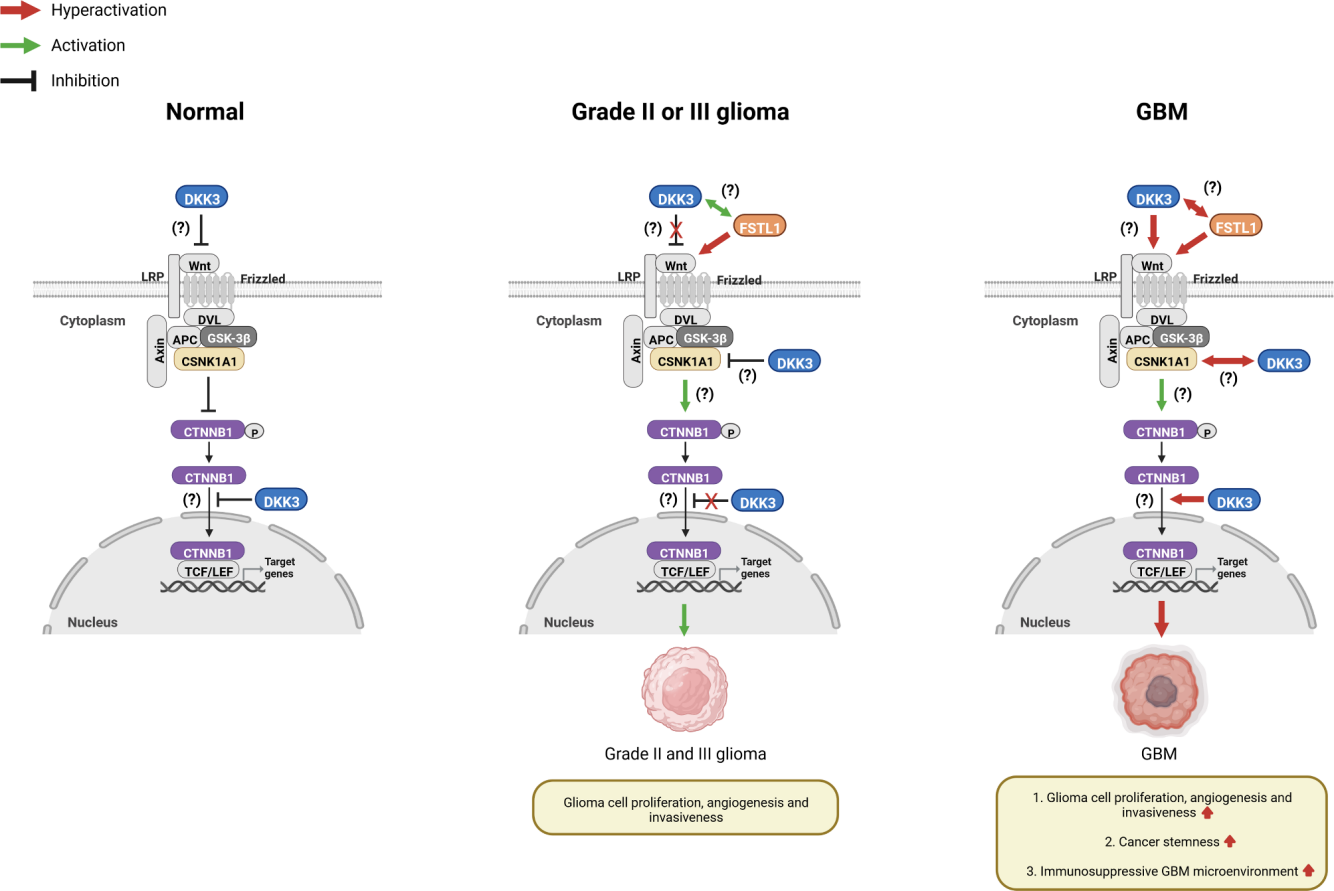
Schematic illustrations of possible roles of DKK3 in normal and grade II or III glioma and grade IV GBM: DKK3 may suppress the Wnt/β-catenin signaling pathway under normal cell conditions. However, under grade II or III glioma conditions, the function of DKK3 to suppress Wnt/β-catenin signaling may be inhibited. DKK3 may positively interact with FSTL1, leading to activation of the Wnt/β-catenin signaling pathway in grade II or III glioma. CSNK1A1 may change its role in degradation of β-catenin and instead activate β-catenin signaling in the glioma environment. Although DKK3 may be associated with activation of Wnt/β-catenin in LGG, it simultaneously may also inhibit CSNK1A1, leading to inhibition of β-catenin signaling. On the other hand, in the environment of GBM, the role of DKK3, which was found to have two contradictory roles in grade II or III glioma, may be altered again. In GBM, DKK3 seems to lose its role in suppressing Wnt/β-catenin signaling, which was partially present in LGG, and only play a role in overall hyperactivating the Wnt/β-catenin signaling pathway. This hyperactivation of the Wnt/β-catenin signaling pathway may accelerate GBM cell proliferation, invasiveness, and cell stemness and induce an immunosuppressive GBM microenvironment *APC*, adenomatous polyposis coli; *CSNK1A1*, casein kinase 1 alpha 1; *CTNNB1*, catenin beta 1; *DKK3*, dickkopf Wnt signaling pathway inhibitor 3; *DVL*, dishevelled segment polarity protein; *FSTL1*, follistatin-like 1; GBM, glioblastoma multiforme; *GSK-3β*, glycogen synthase kinase 3 beta; *LEF*, lymphoid enhancer binding factor; LGG, lower grade glioma; *LRP*, LDL receptor-related protein; *TCF*, transcription factor

Based on previously published studies and our current findings, we herein present schematic illustrations of the hypothetical role of DKK3 in glioma progression considering its interaction with CTNNB1, FSTL1, and CSNK1A1, which are involved in the Wnt/β-catenin signaling pathway (Fig. 5).

### Validation of DKK3 expression levels between different grades of glioma tissue samples collected in our hospital

**Fig. 6:**
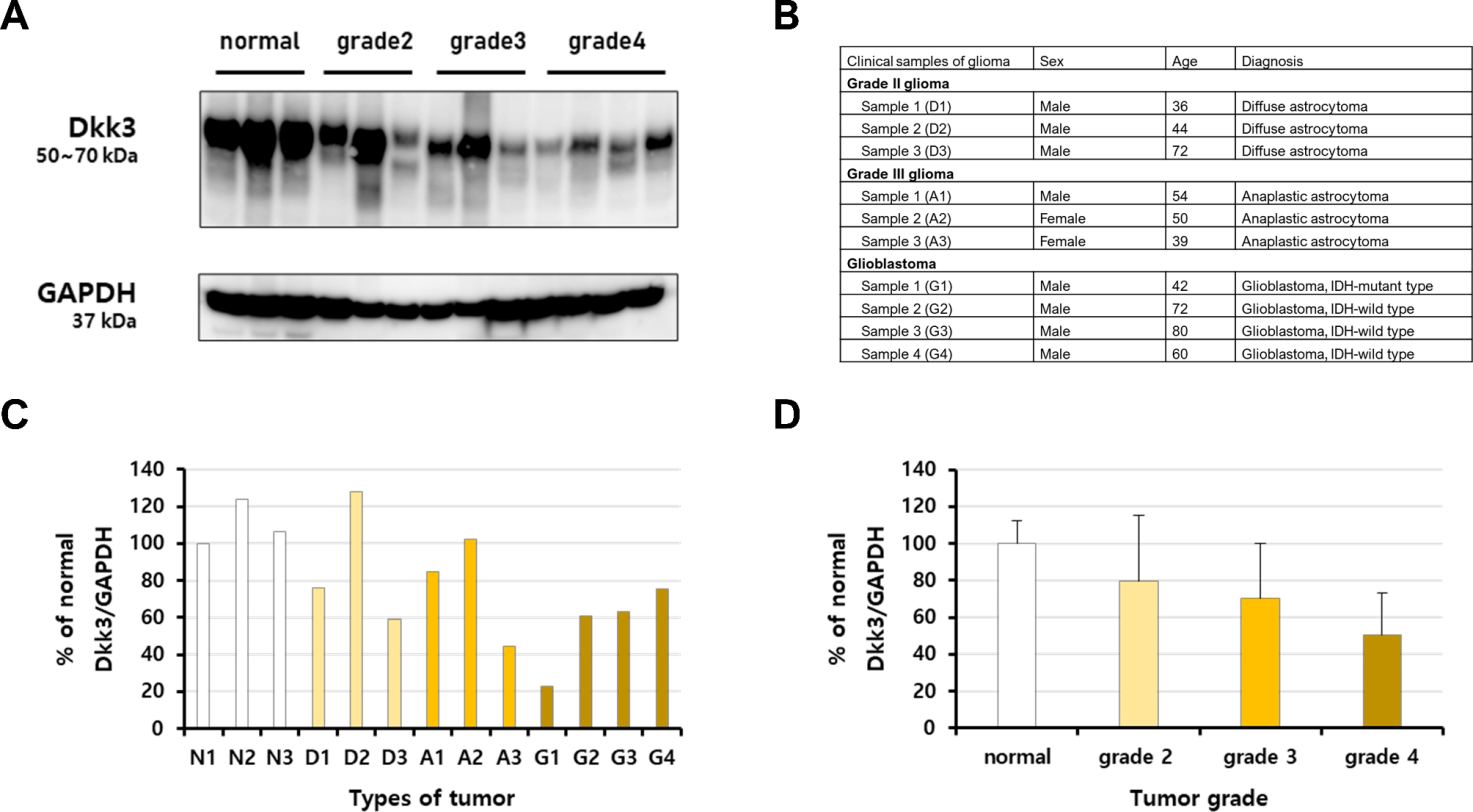
Expression of the DKK3 protein in the normal human brain, grade II glioma, grade III glioma, and grade IV GBM tissues, as observed on western blotting. **(A)** Comparison of DKK3 expression levels between the normal human brain, grade II glioma, grade III glioma, and grade IV GBM by western blotting; **(B)** The information collected regarding the clinical glioma samples collected in our hospital; **(C)** Detailed information on DKK3 expression levels according to each glioma sample; **(D)** Detailed DKK3 expression levels according to tumor grade DKK3, dickkopf Wnt signaling pathway inhibitor 3; GBM, glioblastoma multiforme

**Fig. 7:**
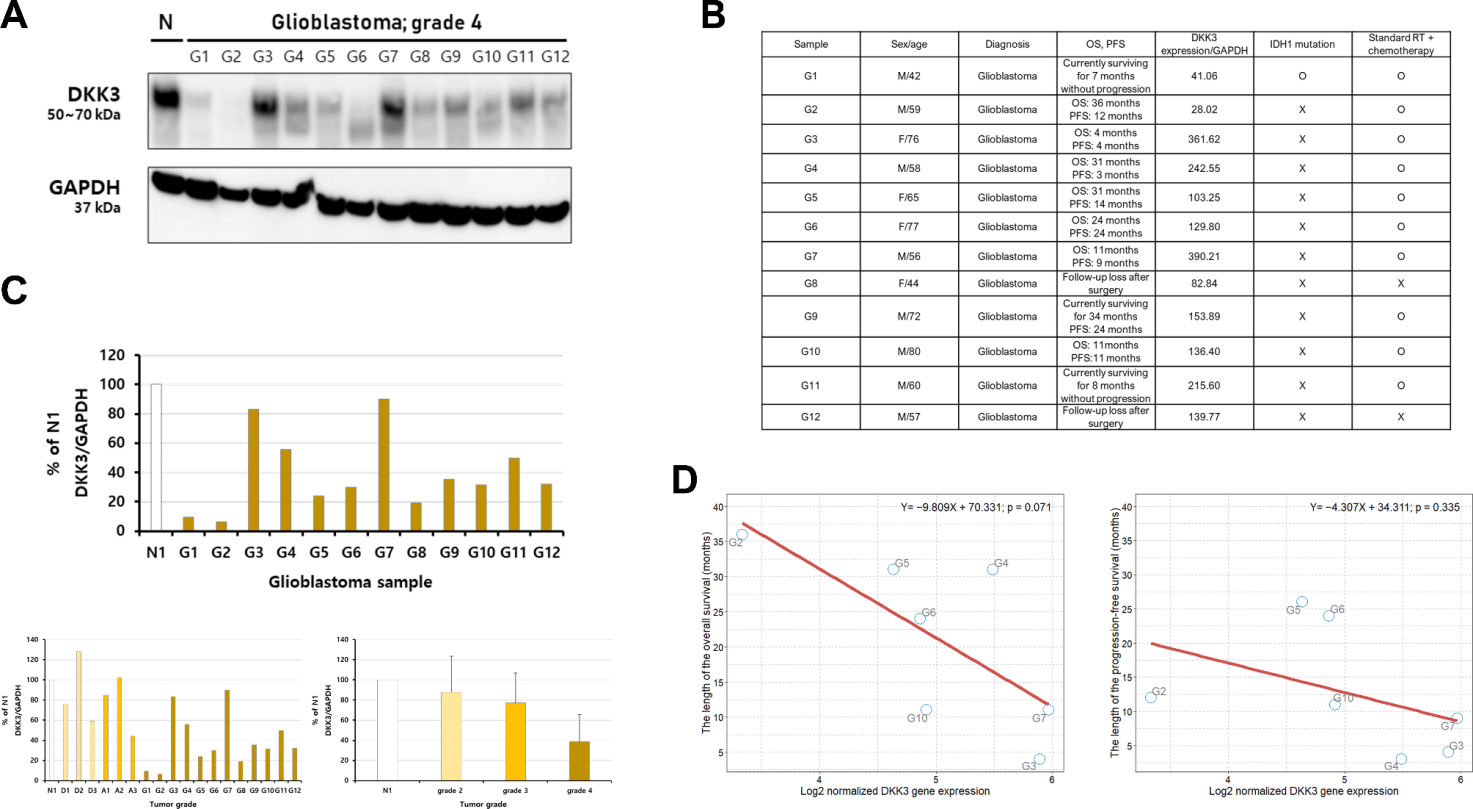
Expression of the DKK3 protein in GBM tissues, as observed on western blotting. **(A)** Comparison of the expression levels of DKK3 between 12 grade IV GBM samples by western blotting; **(B)** The information collected regarding the clinical GBM samples collected in our hospital; **(C)** Detailed information on DKK3 expression levels according to 12 GBM samples and tumor grade; **(D)** Scatterplot with linear regression lines showing the associations between DKK3 expression and OS and PFS rates in the patients from whom the seven GBM samples were collected in our hospital (five samples were excluded from the analysis because three patients are currently living and two were lost to follow-up) DKK3, Dickkopf Wnt signaling pathway inhibitor 3; GBM, glioblastoma multiforme; OS, overall survival; PFS, progression-free survival

We compared DKK3 expression levels in three samples each of the normal human brain, grade II, and grade III gliomas and four samples of grade IV GBM collected in our hospital. Interestingly, we found that the expression of DKK3 was the highest in the normal brain tissues, and that its expression decreased as the grade of glioma increased, similar to the results documented in the TCGA database (Fig. 6). To validate the adverse effects of DKK3 expression on the prognoses of patients with GBM, we performed additional western blotting analyses to examine the differential expression of DKK3 in 12 GBM samples collected in our hospital (Fig. 7A **and B)**. We observed differences in DKK3 expression among the 12 GBM samples; as mentioned above, as the tumor grade increased, the overall expression of DKK3 decreased (Fig. 7C). In addition, on comparing the OS and PFS rates in seven patients with GBM whose OS and PFS assessments were completed, we observed that the OS and PFS rates in patients with GBM tended to decrease as DKK3 expression levels increased, although this result was not statistically significant, likely due to the small sample size (Fig. 7D).

## Discussion

It has been reported that up to 70% of low-grade gliomas transform into high-grade ones within 10 years [15]. This transformation involves changes in many genes and molecular pathways that affect tumor prognosis [12]. Therefore, these differentially expressed genes between low- and high-grade gliomas can be used as biomarkers and therapeutic targets in clinical practice for the diagnosis and treatment of gliomas, potentially improving the prognosis of GBM [15]. In this study, we found significant differences in gene expression profiles between LGG and GBM. We found that *DKK3, CSNK1A1, FSTL1*, and *CTNNB1* were differentially expressed in LGG and GBM.

Overall, we found that significant positive associations between genes belonging to the Wnt/β-catenin pathway were significantly increased in GBM compared with LGG. We also observed that as the grade of glioma increased, DKK3 expression showed a tendency to be more strongly positively correlated with the expression of CTNNB1, FSTL1, and CSNK1A1, which are related to the Wnt/β-catenin pathway. In addition, according to our findings, DKK3 was not associated with immunosuppression in LGG but was associated with downregulation of immune responses in GBM tissue. Furthermore, in LGG, we observed significant positive associations between DKK3 expression and the cell fractions of CD8 + T cells and memory B cells. In contrast, in GBM, a significant negative relationship was shown between DKK3 expression and the CD8 + T-cell and memory B-cell fractions. DKK3 is known suppress CD8 + and CD4 + T-cell-mediated responses [26–28]. Therefore, we hypothesized that high DKK3 expression may be associated with an immunosuppressive GBM microenvironment and that the association between DKK3 expression and immune responses may be altered according to the grade of glioma. Although the finding was not statistically significant, our study showed that patients with LGG in the high and low DKK3 expression groups showed slightly improved OS and PFS rates compared with those in the moderate DKK3 expression group. However, as previously described, higher DKK3 expression in GBM was significantly associated with an increased risk of mortality and disease progression compared with lower DKK3 expression [11]. Although the sample size was small, additional validation analyses were performed using glioma samples collected in our hospital.

Therefore, considering these findings, we hypothesized that the role of DKK3 in the Wnt/β-catenin pathway might be different between LGG and GBM (Fig. 5). The Wnt signaling inhibitor DKK3 is known as a potential key player in tumor suppression, and the expression of DKK3 is found to be frequently downregulated in almost any cancer entity [29]. Therefore, we believe that DKK3 somewhat suppresses the Wnt/β-catenin signaling pathway under normal cell conditions. However, whether DKK3 promotes or inhibits the Wnt/β-catenin signaling pathway remains highly controversial [29–34]. Interestingly, our findings suggest that two seemingly contradictory roles of DKK3 appear to coexist with respect to the Wnt/β-catenin pathway in LGG. According to our findings, under grade II or III glioma conditions, the function of DKK3 to suppress Wnt/β-catenin signaling seems to be somewhat inhibited. Furthermore, based on our findings, DKK3 may positively interact with FSTL1, which is known to activate the Wnt/β-catenin pathway and is involved in tumorigenesis [35, 36]. This may activate the Wnt/β-catenin signaling pathway in grade II or III glioma, leading to glioma proliferation. It is well-documented that the β-catenin destruction complex including glycogen synthase kinase 3 (GSK-3), CSNK1A1, scaffolding protein Axin, and adenomatous polyposis coli (APC), inhibits stabilization of β-catenin, which translocates to the nucleus and binds to transcription factor/lymphoid enhancer binding factor (TCF/LEF) proteins to activate Wnt target gene transcription [37]. However, according to previous studies, GSK3B or CSNK1, which belong to the β-catenin destruction complex, which inhibits activation of β-catenin signaling, can paradoxically induce or aggravate cancer [38–41]. Therefore, based on our findings, we hypothesized that CSNK1A1 may change its role in degrading β-catenin and rather activate β-catenin signaling in the glioma environment. However, according to our results, although DKK3 may be associated with activation of Wnt/β-catenin in LGG, it simultaneously may also inhibit CSNK1A1, leading to inhibition of β-catenin signaling. Therefore, we hypothesized that these two contradictory roles of DKK3 related to the Wnt/β-catenin pathway (either activating or inhibiting β-catenin) in LGG may have a dual impact on the prognosis of glioma by causing proliferation of glioma cells while maintaining the immune microenvironment of glioma. Although this finding was not statistically significant, the low and high DKK3 expression groups tended to have a longer survival time or disease-free period than the moderate DKK3 expression group, supporting this hypothesis (Fig. 4A). On the other hand, in the environment of GBM, the role of DKK3, which had two contradictory roles in grade II or III glioma, may change again. Based on our study results, in GBM, DKK3 appears to lose its role in suppressing Wnt/β-catenin signaling, which was partially present in LGG, and only play a role in overall hyperactivating the Wnt/β-catenin signaling pathway. This hyperactivation of the Wnt/β-catenin signaling pathway may accelerate GBM cell proliferation, invasiveness, and GBM cell stemness and induce an immunosuppressive GBM microenvironment. Therefore, we hypothesized that the effect of DKK3 on hyperactivation of the Wnt/β-catenin pathway in GBM may be critical enough to significantly affect mortality and disease progression in patients with GBM.

In this study, we unexpectedly found that the expression of DKK3 was higher in LGG than in GBM. We do not know the underlying causes for the decreased expression of DKK3 in GBM compared with LGG, which has a significant prognostic effect on patients with GBM. We reported higher expression of DKK3 in GBM than in LGG in our recent study [11]. However, in that study, we briefly compared DKK3 expression between LGG and GBM. The small number of patients for which DKK3 expression was compared between LGG and GBM in that study was sampled from the TCGA’s pancanatlas, which included only patients with all mRNA expression information for more than 20,000 genes, and these patients were not analyzed among the cohort of the current study [42]. As described in the Methods section, the same 525 patients with GBM from the TCGA database in our recent study were also included in this study. Furthermore, the mean DKK3 expression level in patients with GBM in our previous study was the same as that among patients with GBM in this current study (shown in Supplementary Fig. 1B of our recent study) [11].

The confusion may be caused by the decreasing DKK3 expression with increasing tumor grade, resulting in DKK3 appearing to be a tumor suppressor gene; however, in GBM, it acts as an oncogene because increased expression of the gene is associated with poor patient prognosis. As shown in Fig. 1C, when analyzed using TCGA data, we found that contrary to our expectations, DKK3 expression levels decreased with increasing tumor grade. However, we published in a recent paper that DKK3 acts as an oncogene in GBM, and its higher expression is associated with poor patient outcomes (Fig. 4B) [11]. As shown in Fig. 3, we discovered that DKK3 acts as a tumor suppressor gene in LGG by increasing tumor microenvironmental immunity whereas, as an oncogene in GBM by inducing an immunosuppressive microenvironment. Therefore, we hypothesized that DKK3 may play different roles in LGG than it does in GBM.

As described above, whether DKK3 promotes or inhibits the Wnt/β-catenin signaling pathway remains controversial [34]. Therefore, unlike DKK 1, 2, and 4, there is still some debate about whether DKK3 is an oncogene or a tumor suppressor gene [30–33]. Therefore, we speculate if the reason why the role of DKK3 has not yet been established, unlike DKKs 1, 2, and 4, is because the role of DKK3 in Wnt/β-catenin signaling may change depending on the tumor grade or tumor type, as shown in our results. Our results suggest that in LGG, unlike in GBM, DKK3 may also act as a tumor suppressor gene to some extent in relation to Wnt/β-catenin signaling. We illustrate this hypothesis in Fig. 5.

Therefore, we believe that it is difficult to conclude if DKK3 is a tumor suppressor gene or not solely based on the fact that its expression is high in LGG and low in GBM. Rather, our results suggest that DKK3 acts as a tumor suppressor gene in LGG by increasing tumor microenvironmental immunity, but as an oncogene in GBM by decreasing it. In addition, when we analyzed the prognosis of patients with GBM based on the expression of DKK3, it seems to act as an oncogene, unlike its role in LGG. Therefore, as shown in Fig. 5, we believe that DKK3 may change its role in Wnt/β-catenin signaling depending on the type or grade of the tumor. Moreover, we believe that in cancer, DKK3 can act as both a tumor suppressor gene and an oncogene. Furthermore, we consider the pathophysiology associated with DKK3 to be more complex than previously established. More studies will be needed to prove this in the future.

This study has several limitations. First, we obtained all clinical and mRNA expression data from the TCGA database, which is retrospective in nature; thus, further prospective studies are necessary to validate the results. However, we have presented all the TCGA data used in this study as supplementary data; therefore, researchers will be able to transparently verify our results. Second, the fraction of immune cells was calculated using in silico flow cytometry-based analysis of immune-related genes. There may be a difference in the real number of immune cells. Third, the current findings should also be verified further through additional experimental analyses using methods other than western blotting. Further in vitro and in vivo studies are therefore warranted. Fourth, due to the nature of the TCGA data, there were missing clinical and mRNA expression data. This may have affected the results of the statistical analysis in the study. Fifth, because of the nature of TCGA data, it was difficult to classify gliomas based on their molecular subtypes.

In conclusion, we investigated the possible role of DKK3 associated with Wnt/β-catenin signaling in LGG and GBM using a large-scale, open database. According to our findings, DKK3 appears to play two contradictory roles, either activating or inhibiting β-catenin in LGG. However, in GBM, DKK3 only induces hyperactivation of the Wnt/β-catenin pathway by interacting with genes related to the Wnt/β-catenin pathway, which seems to have a significant effect on mortality and disease progression in patients with GBM. Although our findings need to be validated in the future, we believe that they may contribute to improving understanding of the mechanisms underlying the pathophysiology of glioma.

## Electronic supplementary material

Below is the link to the electronic supplementary material.

Supplementary Material 1

Supplementary Material 2

## Data Availability

The datasets generated during and/or analysed during the current study are available from the corresponding author on reasonable request.
