# Supplementary material for: DKK3 expression is associated with immunosuppression and poor prognosis in glioblastoma, in contrast to lower-grade gliomas
Source: BMC Neurol. 2023 May 6;23:183. doi: 10.1186/s12883-023-03236-0 (PMC10163766; doi:10.1186/s12883-023-03236-0)
Supplement: Supplementary file 2 — Supplementary Material 2 [file 12883_2023_3236_MOESM2_ESM.pdf]

Supplementary Material

DKK3 expression is associated with immunosuppression and poor prognosis in glioblastoma, in contrast to lower-grade gliomas

Myung-Hoon Han, Jeong Min Baek, Kyueng-Whan Min, Jin Hwan Cheong, Je Il Ryu, Yu Deok Won, Mi Jung Kwon, Seong-Ho Koh

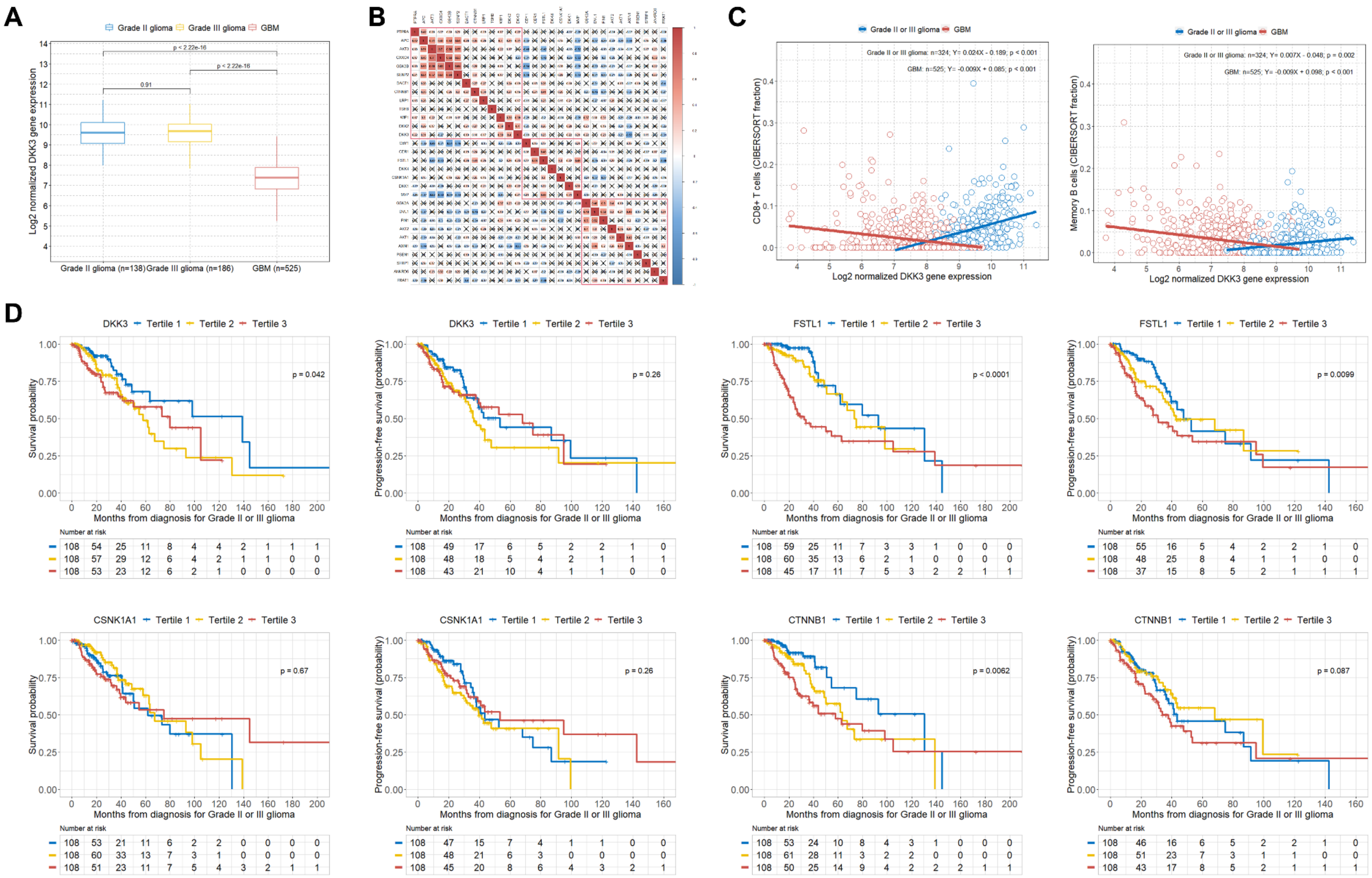
A
Grade II glioma
Grade III glioma
GBM
Log2 normalized DKK3 gene expression
p < 2.22e-16
0.91
Grade II glioma (n=138)
Grade III glioma (n=186)
GBM (n=525)
B
C
Grade II or III glioma
GBM
Grade II or III glioma: n=324; Y= 0.024X - 0.189; p < 0.001
GBM: n=525; Y= -0.009X + 0.085; p < 0.001
CD8+ T cells (CIBERSORT fraction)
Log2 normalized DKK3 gene expression
Grade II or III glioma: n=324; Y= 0.007X - 0.048; p = 0.002
GBM: n=525; Y= -0.009X + 0.098; p < 0.001
Memory B cells (CIBERSORT fraction)
D
DKK3 Tertile 1 Tertile 2 Tertile 3
FSTL1 Tertile 1 Tertile 2 Tertile 3
CSNK1A1 Tertile 1 Tertile 2 Tertile 3
CTNNB1 Tertile 1 Tertile 2 Tertile 3
Survival probability
Progression-free survival (probability)
Months from diagnosis for Grade II or III glioma
Number at risk
p = 0.042
p = 0.26
p < 0.0001
p = 0.0099
p = 0.67
p = 0.26
p = 0.0062
p = 0.087

Supplementary Fig. 1. Additional analysis excluding patients with oligodendroglioma from the LGG group. (A) Boxplots showing the differences in DKK3 expression levels according to the WHO grade of glioma; (B) Pearson correlation coefficients and significance levels calculated between Wnt/β-catenin pathway-related genes in WHO grades II and III gliomas; (C) Scatterplots with linear regression lines showing the associations between DKK3 expression and the CD8+ T-cell and memory B-cell fractions according to WHO grades II or III gliomas and GBM; (D) Kaplan–Meier curves showing the OS and PFS rates according to DKK3, FSTL1, CSNK1A1, and CTNNB 1 tertiles in WHO grades II or III gliomas.

DKK3, dickkopf Wnt signaling pathway inhibitor 3; GBM, glioblastoma multiforme; OS, overall survival; PFS, progression-free survival

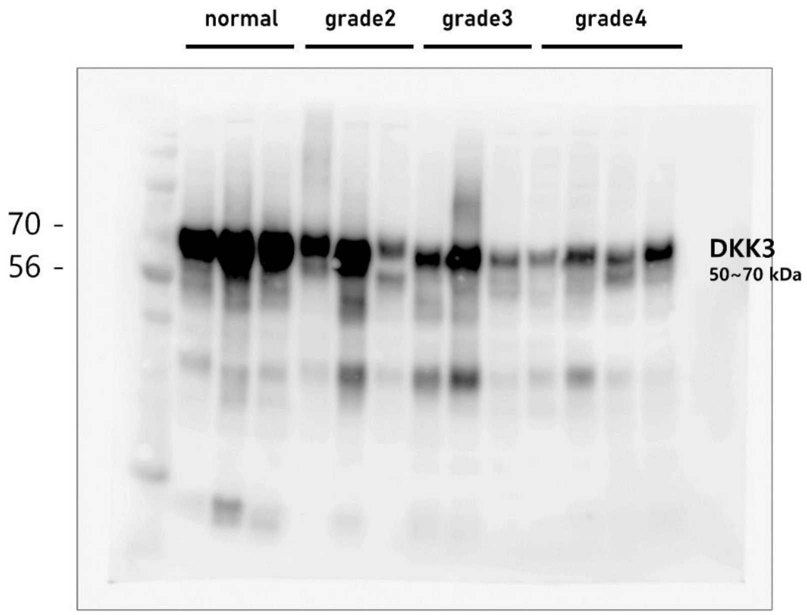

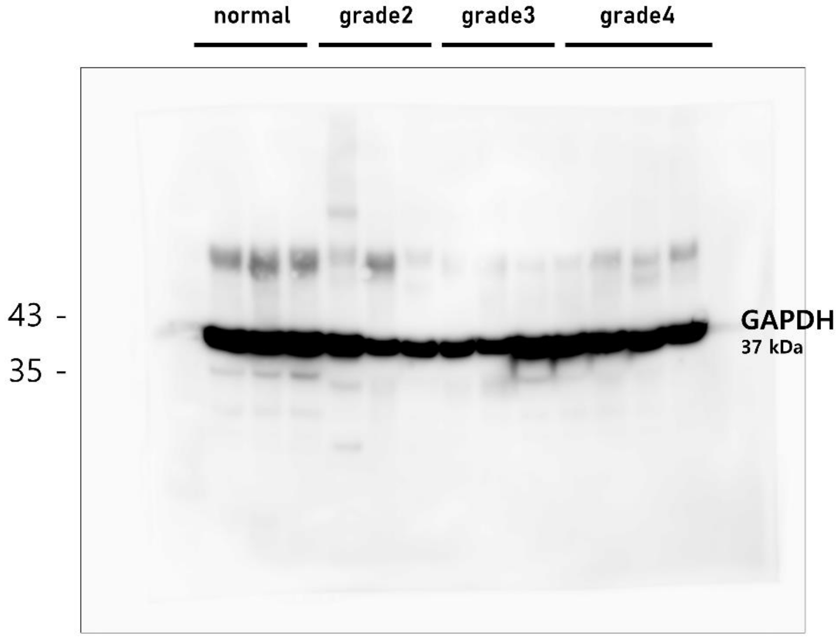

Supplementary Fig. 2. Full-length blots/gels of Fig. 6A

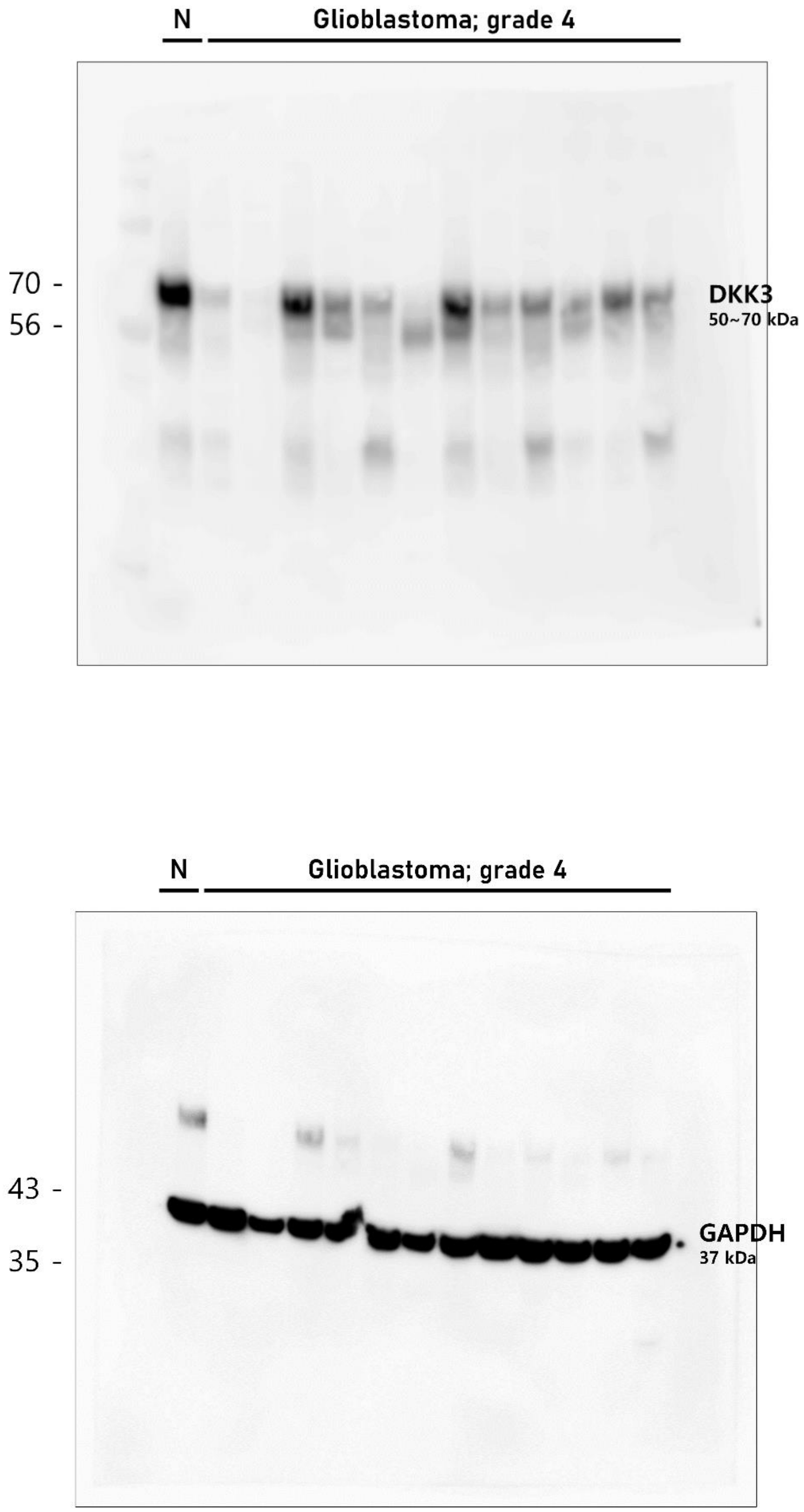

Supplementary Fig. 3. Full-length blots/gels of Fig. 7A
